# Acute cardiovascular responses to slow and deep breathing in normotensive non-pregnant and pregnant women

**DOI:** 10.3389/fnetp.2026.1913383

**Published:** 2026-10-14

**Authors:** Malika Felton, Stephanie Grigsby, Vanora A. Hundley

**Affiliations:** 1 Centre for Midwifery & Women’s Health, Bournemouth University, Bournemouth, United Kingdom; 2 University Hospitals Dorset, NHS Foundation Trust, Bournemouth, United Kingdom

**Keywords:** blood pressure, network physiology, pregnancy, respiratory, respiratory sinus arrhythmia, slow and deep breathing

## Abstract

**Objective:**

To compare the acute cardiovascular responses of non-pregnant and pregnant women to slow and deep breathing (SDB) at a range of breathing frequencies. Participants: Forty-one women (23 non-pregnant and 18 pregnant).

**Methods:**

Participants completed five different breathing conditions in a randomised order (5-min duration); spontaneous breathing (S*f*_*r*_), dynamic SDB breathing frequency (D*f*_*r*_), and three fixed breathing frequencies of 4 breaths^.^ min^−1^ (4F*f*_*r*_), 6 breaths. min^−1^ (6F*f*_*r*_), and 8 breaths. min^−1^ (8F*f*_*r*_). Main Outcome Measures: Non-invasive beat-to-beat arterial blood pressure, heart rate and respired airflow were monitored continuously.

**Results:**

The maximum amplitude of respiratory sinus arrhythmia (RSA) during SDB was 2.1 times higher than during spontaneous breathing (S*f*_*r*_) for both groups, maximising at 6 breaths^.^ min^−1^ (0.25s ± 0.1 non-pregnant, 0.15s ± 0.1 pregnant). Although absolute RSA was significantly lower in pregnant women during spontaneous breathing (S*f*_*r*_; 0.07s vs. 0.12s non-pregnant), SDB increased RSA to the same level as non-pregnant women’s spontaneous breathing levels. In both groups, the amplitude of blood pressure oscillations (both systolic and diastolic blood pressure) increased as breathing frequency declined, reaching a peak at 4 breaths^.^ min^−1^ (4F*f*_*r*_), which was up to 4.5 times higher (14.3%, 10 mmHg) than during spontaneous breathing (S*f*_*r*_).

**Conclusion:**

Cardiovascular responses maximised at ≤6 breaths^.^ min^−1^ and acute haemodynamic responses to SDB are similar in pregnant and non-pregnant women, despite absolute differences under spontaneous breathing conditions. If blood pressure oscillations underpin the anti-hypertensive effect of SDB, the data indicate that these can be amplified in pregnant women at frequencies between 4 and 6 breaths^.^ min^−1^.

## Introduction

Gestational hypertension occurs in up to 15% of pregnancies (James and Nelson-Piercy, 2004) and causes an increased risk of obstetric complications (Scantlebury et al., 2013). It is defined as high blood pressure presenting after 20 weeks of pregnancy, which was not present prior to pregnancy (NICE: National Institute for Health and Care Excellence, 2019), and while pharmaceutical treatments exist, the only cure is to give birth. Since many women have an aversion to medication in pregnancy (Twigg et al., 2016), pregnant women are highly motivated to adhere to and undertake non-pharmacological interventions (Adams et al., 2009). Slow and deep breathing (SDB) is recommended by the American Heart Association as an adjunctive treatment for hypertension in non-pregnant populations (Brook et al., 2013) and a recent meta-analysis of studies on SDB in primary hypertension found that following daily practice of SDB reductions of up to 5.26 mmHg for systolic blood pressure (SBP) and 2.97 mmHg for diastolic blood pressure (DBP) were observed (Chaddha et al., 2019). Given that the aetiology of gestational hypertension has also been linked to high breathing frequencies (Fischer and Voss, 2014) and dysfunctional breathing (Jerath et al., 2009), SDB offers a potential effective treatment for uncomplicated gestational hypertension (Felton et al., 2021). Alternative breathing based exercises such as mindfulness and diaphragmatic breathing without fixed SDB frequencies have also showed promise in reducing blood pressure in women with pre-eclampsia (Kılıçlı and Simge, 2025; Ibrahim et al., 2024).

However, pregnancy itself induces a series of cardiovascular adaptations, which may subsequently change the acute response to SDB, compared with those of non-pregnant women. For example, during pregnancy, baseline cardiovascular measures such as heart rate, stroke volume and cardiac output are increased above non-pregnant levels (Soma-Pillay et al., 2016). It is possible that these changes in baseline cardiovascular function may influence the acute cardiovascular response to SDB. Additionally, the health benefits and reductions in blood pressure (BP) associated with SDB are suggested to be related to diaphragmatic breathing (Gerritsen and Band, 2018), however during pregnancy the diaphragm is forced upwards by as much as 5 cm (Elkus and Popovich, 1992), which may limit its mobility and the ability to perform SDB and/or achieve any associated health benefits.

Therefore, prior to undertaking an intervention using SDB as a treatment for gestational hypertension there is a need to characterise and understand the acute responses to SDB of pregnant women and whether these differ from non-pregnant women. A review of international guidance for the management of hypertensive disorders of pregnancy found non-pharmacological interventions lacked evidence supporting the potential benefits, which lead to a lack of clear guidance in this area (Danielli et al., 2025), so it is important to provide evidence to support any future SDB intervention for gestational hypertension. Firstly, normative and baseline data is needed as a comparison before moving forward to investigate SDB with women who develop gestational hypertension. Additionally, recent research suggests that when investigating SDB a multi-parametric approach is required, and investigating inter- and intra-breath phase cardiovascular oscillations is essential (Felton et al., 2026). It is therefore important to take a Network Physiology approach and consider the maternal physiological system as a network of physiological interactions and not as separate independent systems (Ivanov, 2021), with physiological systems interacting and synchronising to respond to SDB. Consequently, the aim of this study was to compare the acute cardiovascular responses of non-pregnant and pregnant women to SDB at a range of breathing frequencies.

## Materials and methods

All experiments conformed to the Declaration of Helsinki and the experimental protocol was approved by Bournemouth University’s Research Ethics Committee (ID 19148 and 22930). Written informed consent was obtained from all participants prior to participating in the study.

## Participants

Forty-one women participated in the study: 23 healthy non-pregnant women and 18 healthy pregnant women. All participants were of reproductive age as defined by the World Health Organization (15–49 years) (World Health Organization, 2006) and all pregnant women were over 20-weeks gestation. Participants were recruited from the local student and staff population at a Higher Education Institution, and by using local antenatal and maternal groups including social media. All pregnant women were nulliparous and were carrying singleton pregnancies. Participants diagnosed with any cardiovascular or respiratory disease were excluded, as were smokers and women who vaped. All participants were normotensive at the time of data collection and the pregnant women submitted regular BP measurements until birth to confirm they did not subsequently develop high BP during their pregnancy.

Participants attended one session at the Cardiorespiratory Research Laboratory at Bournemouth University. Prior to the data collection session participants refrained from eating for 2 h and from caffeine, strenuous exercise and alcohol for 12 h. Average laboratory conditions (temperature, pressure, humidity) during data collection were 24.0 ± 3.2 °C, 992.6 ± 13.5 h Pa, 42.6% ± 10.6%.

## Slow and deep breathing protocol

A recent study (Felton et al., 2026) found that SDB increases the amplitude of respiratory sinus arrhythmia (RSA) and BP oscillations with declining breathing frequency, with maximal amplitudes occurring at 6 breaths^.^ min^−1^ (0.1 Hz). It was speculated that the oscillations in BP elicited by SDB might underpin its anti-hypertensive effect. However, 6 breaths^.^ min^−1^ was the lowest breathing frequency assessed, and it is possible that even lower breathing frequencies might increase the amplitude of BP oscillations further. To date, previous studies that have compared cardiovascular responses to SDB at a range of frequencies have done so using a SDB protocol that reduced breathing frequency dynamically, with only short durations at each individual SDB frequency (Zhang et al., 2024; Anderson et al., 2009). The present study therefore undertook a systematic characterisation of the acute cardiovascular responses to a range of steady-state SDB frequencies, including 4 breaths^.^ min^−1^.

Participants completed five breathing conditions in a randomised order; spontaneous breathing (S*f*_*r*_), 4 (4F*f*_*r*_), 6 (6F*f*_*r*_), and 8 (8F*f*_*r*_) breaths^.^ min^−1^, and a dynamic frequency using an optimisation algorithm (D*f*_*r*_), which maximised respiratory sinus arrhythmia (RSA). Randomisation was conducted using a random number generator (www.randomizer.org). All breathing conditions were 5-min in duration with a 5-min break of normal breathing between each measurement. All SDB conditions were delivered by an app, which delivers either fixed breathing frequencies (4F*f*_*r*_, 6F*f*_*r*_, 8F*f*_*r*_) or uses a novel, bespoke algorithm to deliver a personalised dynamic frequency (D*f*_*r*_). The algorithm maximised cardiovascular perturbations, using the amplitude of RSA as the controlled variable. Changes in RSA were measured from a finger sensor (photoplethysmography), connected via the headphone socket of an iPad. The app displayed visual feedback on an iPad screen to guide breathing; user’s inhale when the dome graphic rises and exhale when the dome falls (Figure 1).

**FIGURE 1:**
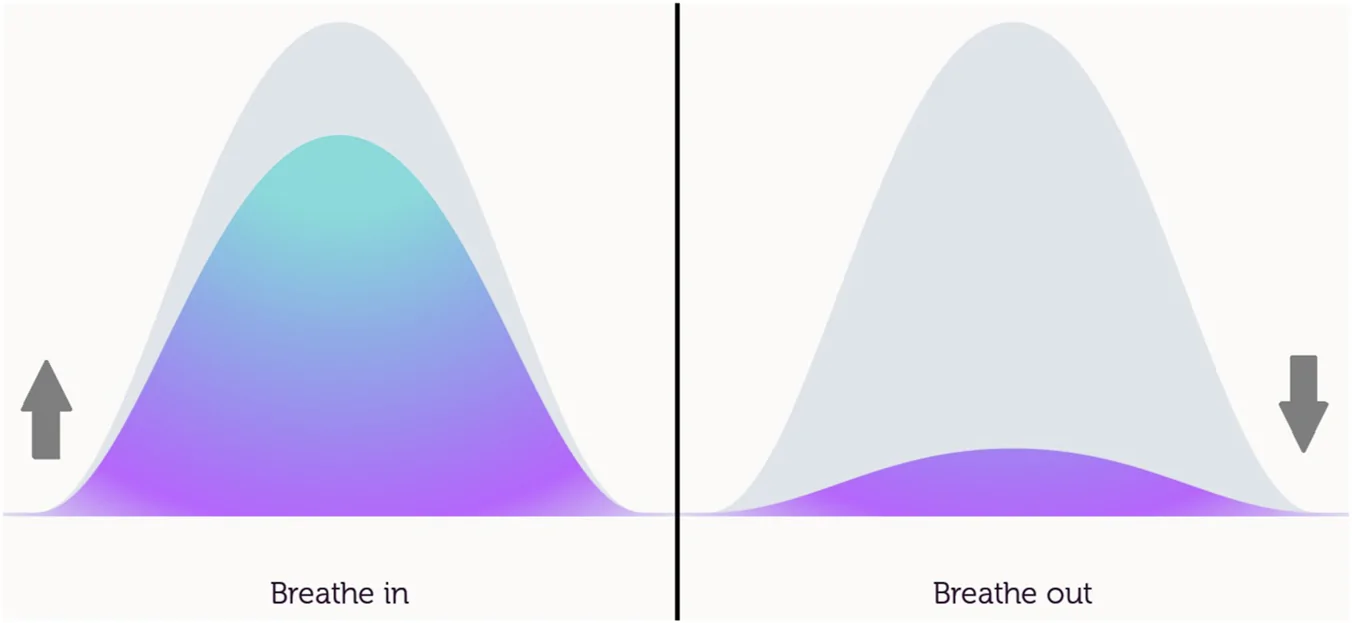
Screenshots of the app graphic. *N.B: Arrows do not appear on app but are shown here to display the direction of graphic movement.* From *Acute cardiovascular responses to slow and deep breathing in normotensive men and women* [Figure 1]. (Felton et al., 2026) CC by 4.0.

Breathing frequencies of 4 and 8 breaths^.^ min^−1^ were chosen to include frequencies either side of the widely reported ‘optimal’ breathing frequency of 6 breaths^.^ min^−1^ (Cullins et al., 2013; Russo et al., 2017). Following completion of the protocol, the pregnant participants were asked which breathing condition they felt most comfortable breathing at and would choose to use if they were asked to continue undertaking the breathing exercise daily until birth.

## Data measurement and acquisition

During each breathing condition, respiratory airflow, electrocardiogram (ECG) and arterial BP were monitored continuously. Participants were seated in an upright position, at an approximate angle of 60°. Respired flow rate was measured continuously using a heated pneumotachograph (Model 3700, Hans Rudolph Inc., Kansas, United States of America), connected to a flow measurement system (RSS 100-HR, Hans Rudolph Inc., Kansas, United States of America) while participants wore an oronasal mask (Oro Nasal 7450 V2 Mask, Hans Rudolph Inc., Kansas, United States of America).

A 3-lead ECG measured heart rate continuously, whilst non-invasive beat-to-beat arterial BP was obtained using finger photoplethysmography (Finapres NOVA, Finapres Medical Systems, Netherlands). Finapres derived arterial BP was calibrated using a brachial cuff prior to and halfway through data collection. Analogue outputs from the Finapres NOVA and the flow meter were sampled continuously at 250 Hz via an analogue to digital converter (NI USB-6218 BNC, National Instruments Inc.) and captured using acquisition and analysis software (LabView 2015; National Instruments, Inc.). The LabView software corrected for the 4 s delay between the Finapres NOVA output and the respiratory output. Stroke volume (SV) was calculated using the Modelflow method (Wesseling et al., 1993) by the Finometer. Total peripheral resistance (TPR) was calculated as mean arterial pressure divided by cardiac output (Q̇). Pulse wave analysis (PWV) was calculated as the distance between sternal notch and Finometer finger cuff divided by pulse transit time (Hansen, 2010). Pulse transit time was calculated as the time delay between the peak of the R wave of the ECG and the peak of the pressure pulse recorded at the finger, with the peaks detected using the LabView software.

Stretch stature was measured using a stadiometer (SECA 213, Germany) and body mass was recorded in minimal clothing using calibrated electronic scales (SECA 804, Germany).

## Data analysis

The LabView software calculated and analysed variables beat-by-beat and breath-by-breath, including the minimum, maximum and mean values for each inspiration and expiration breath phase. Data were averaged across each 5-min breathing condition.

Values are expressed as mean ± SD unless stated otherwise. Statistical analysis was undertaken using SPSS Statistics 29 (IBM Corp.) and JASP (0.19.3). After normality was confirmed for variables (Shapiro Wilk) repeated measures ANOVA with planned pairwise comparisons using Bonferroni corrections were used. Between group (pregnant and non-pregnant) comparisons used independent samples t-tests. Reported p values are those following adjustment for repeated comparisons. To examine relationships between variables, Spearman’s rank-order correlation analyses were conducted, after the data violated normality assumptions (Shapiro Wilk). For all analyses, P was set at 0.05.

Respiratory sinus arrhythmia (RSA) was calculated using two methods 1) the difference between the average heart rate (*f*_*c*_) during inhalation (*f*_*c*_i) and exhalation (*f*_*c*_e) for every breath (*f*_*c*_Δ); 2) the difference in maximum and minimum beat-to-beat intervals (RR) during inspiration and expiration respectively for every breath (RSA). *F*_*c*_Δ is also known as RespHRV (Menuet et al., 2025). RSA is a variable calculated to determine the amplitude of heart rate rhythms using the ‘peak-valley’ method, which was also used to analyse all variables including BP in the present study.

Calculated parameters and their derivations are displayed schematically using a sinewave in Figure 2 (with corresponding calculation numbers). Inter-breath phase indices (Δ) were quantified as the difference between mean inspiration (i) and mean expiration (e) values for all variables (calculation 4). Peak-valley (PV) indices were calculated as maximum minus minimum values during inspiration (Δi: calculation 6) and expiration (Δe: calculation 5). Inter-breath phase PV indices (ΔPV) were calculated using maximum inspiration minus minimum expiration, or minimum inspiration minus maximum expiration, dependent on which calculation gave the largest difference. Calculation 7 shows an example using the calculation maximum inspiration (peak) minus minimum expiration (valley). The PV indices were also calculated irrespective of the breath phase in which they occurred, and are referred to herein as peak-valley breath phase independent calculations (ΔPV_Ind; not shown in Figure 2).

**FIGURE 2:**
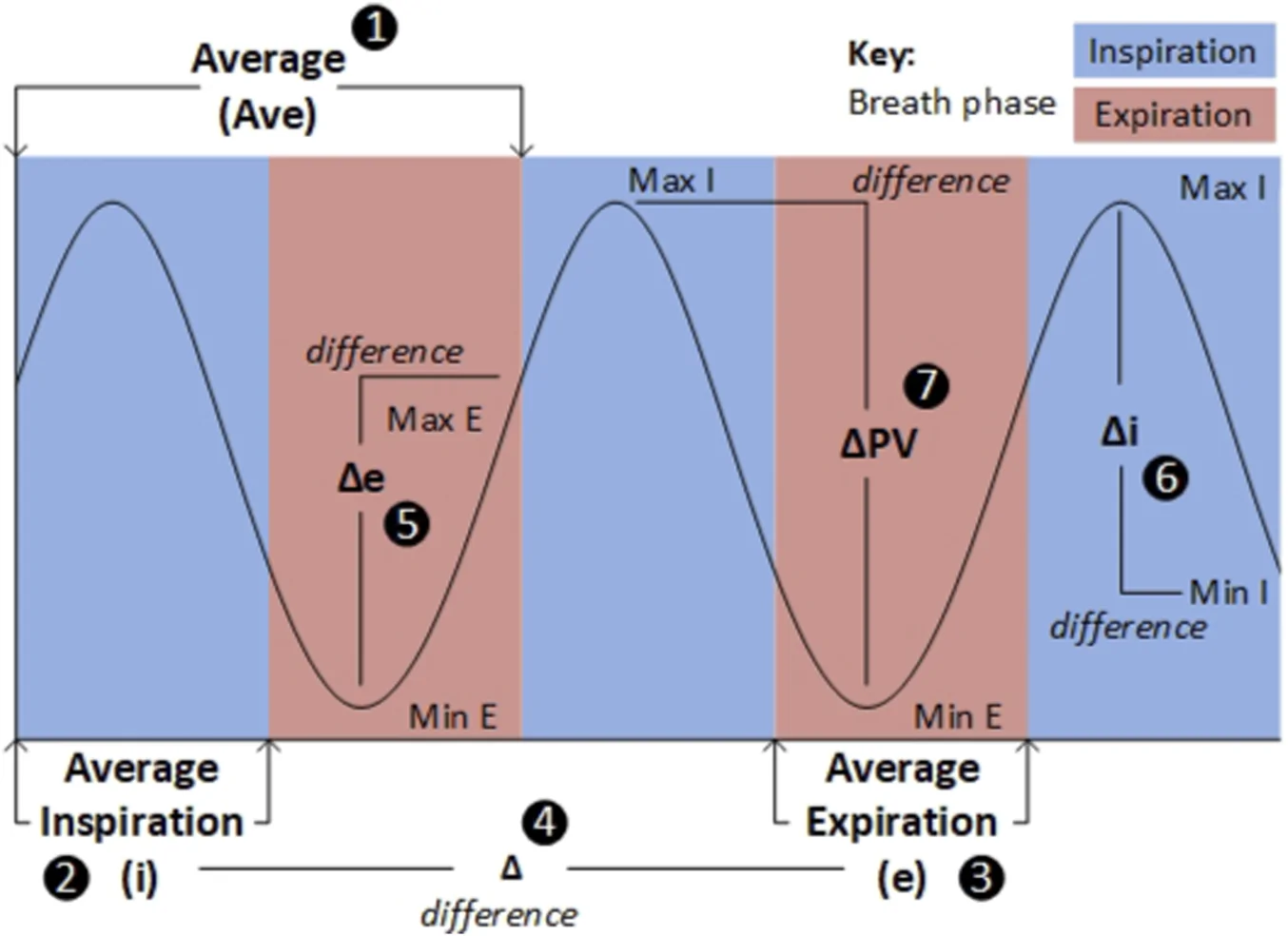
Calculations for example cardiovascular variable plot. E = Expiration breath phase; I = Inspiration breath phase. (1) Ave = average of whole breath; (2) i = average inspiration; (3) e = average expiration; (4) Δ = i minus e (average inspiration minus average expiration); (5) Δ e = Max E minus Min E; (6) Δ i = Max I minus Min I; (7) Δ PV = max I minus min E (note Δ PV calculation varies and can be Min I minus Max E depending on which calculation provides largest difference). From Acute cardiovascular responses to slow and deep breathing in normotensive men and women [Figure 2]. Felton et al. (2026) CC by 4.0.

## Results

Data were collected from 41 participants. Six participants were excluded from the analysis; three due to technical errors in the measurement of respiratory airflow, two because the participant failed to adhere to the prescribed breathing condition and one due to failure of the acquisition system to save the signal data. Consequently, data analysis was performed on data from 18 non-pregnant women and 17 pregnant women. The main findings related to inter- and intra-breath phase analysis are presented below, but the mean data for all variables are presented in the Supplementary Material.

There were no significant differences in age, stature, systolic blood pressure (SBP) or diastolic blood pressure (DBP) between non-pregnant and pregnant participants at baseline (Table 1). Mass was significantly greater (28%; p < 0.001) in pregnant women, accounted for by the growing fetus.

**TABLE 1:** Participant characteristics.

Participant characteristics	Non-pregnant	Pregnant	*P* Value
​	n = 18	n = 17	​
Age (years)	30.1 ± 8.8	32.0 ± 5.4	0.455
Stature (m)	1.66 ± 0.5	1.67 ± 0.8	0.706
Mass (kg)	65.6 ± 10.3	84.1 ± 13.4	<0.001*
Baseline SBP (mmHg)	113.9 ± 9.1	118.2 ± 7.7	0.141
Baseline DBP (mmHg)	68.9 ± 8.0	71.9 ± 7.9	0.265
Gestational age (weeks)	N/A	31.4 ± 5.2	N/A

Systolic blood pressure (SBP), diastolic blood pressure (DBP); *significant difference between groups.

### Respiratory variables

Breathing frequency (*f*_*r*_) was not significantly different between pregnant and non-pregnant women for any breathing conditions, including spontaneous breathing (S*f*_*r*_). Dynamic breathing frequency (D*f*_*r*_) was significantly higher than 6 breaths^.^ min^−1^ for pregnant women (p = 0.016), but not for non-pregnant women (p = 1.000). All other breathing frequencies were significantly different from each other for both groups. Average breathing frequency during S*f*_*r*_ was not correlated with gestational age (r_s_(16) = −0.04, p = 0.881) and neither was the average optimal breathing frequency based on RSA maximisation during D*f*_*r*_ (r_s_ (16) = −0.08, p = 0.765).

Tidal volume was significantly higher for pregnant women during spontaneous breathing (S*f*_*r*_ p = 0.015), but not during any SDB conditions. Duty cycle remained consistent throughout conditions and was not significantly different between groups or between breathing conditions. Full results for respiratory parameters for both groups can be viewed in Table 2.

**TABLE 2:** Respiratory parameters.

Respiratory parameters	Group	Sf_r_	8F*f*_*r*_	Df_r_	6F*f*_*r*_	4F*f*_*r*_
f_r_ (breaths^.^min^−1^)	NP	13.3 ± 2.1^¥¤†§^	8.0 ± 0.0*^¤†§^	6.3 ± 1.1*^¥§^	6.0 ± 0.0*^¥§^	4.0 ± 0.0*^¥¤†^
	P	14.2 ± 2.7^¥¤†§^	8.0 ± 0.1*^¤†§^	7.0 ± 1.1*^¥†§^	6.0 ± 0.0*^¥¤§^	4.0 ± 0.0*^¥¤†^
V_T_ (L)	NP	0.4 ± 0.2^¥¤†§^	0.9 ± 0.4*^†§^	1.0 ± 0.4*	1.1 ± 0.4*^¥^	1.3 ± 0.4*^¥^
	P	0.8 ± 0.5^¤†§^	1.1 ± 0.3^§^	1.1 ± 0.3*^§^	1.5 ± 0.7*	1.6 ± 0.6*^¥¤^
T_I_/T_TOT_	NP	0.42 ± 0.0^¥¤†§^	0.48 ± 0.0*	0.48 ± 0.0*	0.50 ± 0.1*	0.48 ± 0.0*
	P	0.54 ± 0.4	0.52 ± 0.2	0.48 ± 0.1	0.47 ± 0.0	0.56 ± 0.3

Data represent mean ± SD. Non-pregnant (NP) n = 18, Pregnant (P) n = 17. Spontaneous breathing (Sf_r_), fixed breathing frequency of 4 breaths min^−1^ (4F*f*_*r*_), 6 breaths min^−1^.

(6F*f*_*r*_), 8 breaths min^−1^. (8F*f*_*r*_), optimisation algorithm dynamic breathing frequency (Df_r_); breathing frequency (f_r_) in breaths min^−1^, tidal volume (V_T_) in L, duty cycle (T_I_/T_TOT_); Significantly different from Sf_r_ (*), 8F*f*_*r*_ (¥), Df_r_ (¤), 6F*f*_*r*_ (†), 4F*f*_*r*_ (§); P < 0.05.

### Heart rate and respiratory sinus arrhythmia

Peak-valley amplitude changes in heart rate during inspiration (*f*_*c*_Δi) were significantly greater in non-pregnant than pregnant women for all SDB conditions, but not in S*f*_*r*_ (Table 3). Peak-valley amplitude during expiration (*f*_*c*_Δe) and inter-breath phase (*f*_*c*_ΔPV) were significantly higher during all SDB conditions compared with S*f*_*r*_ for both pregnant and non-pregnant participants (Table 3).

**TABLE 3:** Peak-valley differences (±SD) for heart rate (*f*_*c*_ *; beats* ^*.*^ *min* ^*-1*^) and respiratory sinus arrhythmia (RSA).

Peak-valley differences	Group	Sf_r_	8F*f*_*r*_	Df_r_	6F*f*_*r*_	4F*f*_*r*_	Effect of condition *P* value	Group difference P value
f_c_ Δi	NP	5.4 ± 2.2^¥¤†§^	14.4 ± 6.9*	15.5 ± 6.2*	15.5 ± 5.9*	15.9 ± 7.1*	<0.001	0.002
	P	4.5 ± 2.9^¥¤†§^	8.4 ± 5.0*	9.3 ± 5.2*	9.6 ± 5.8*	11.3 ± 5.7*	<0.001	
f_c_ Δe	NP	7.3 ± 3.2^¥¤†^	14.3 ± 6.0*	14.1 ± 8.4*	13.4 ± 6.7*	10.3 ± 8.3	<0.001	0.145
	P	6.1 ± 4.4^¥¤^	10.5 ± 5.2*	9.9 ± 4.6*	9.7 ± 4.0	11.0 ± 6.0	0.004	
f_c_ ΔPV	NP	−6.6 ± 5.1^¥¤†§^	16.1 ± 8.9*^¤†^	20.7 ± 8.5*^¥^	20.6 ± 7.5*^¥^	21.1 ± 9.5*	<0.001	0.044
	P	−3.1 ± 7.4^¥¤†§^	13.4 ± 6.8*	15.3 ± 7.8*	15.1 ± 6.8*	11.2 ± 12.7*	<0.001	
RSA (s)	NP	0.12 ± 0.1^¥¤†§^	0.21 ± 0.1*^¤†^	0.25 ± 0.1*^¥^	0.25 ± 0.1*^¥^	0.25 ± 0.1*	<0.001	0.002
	P	0.07 ± 0.1^¥¤†§^	0.13 ± 0.1*	0.15 ± 0.1*	0.15 ± 0.1*	0.15 ± 0.1*	<0.001	

Data represent mean ± SD. Non-pregnant (NP) n = 18, Pregnant (P) n = 17. Spontaneous breathing (Sf_r_), fixed breathing frequency of 4 breaths min^−1^ (4F*f*_*r*_), 6 breaths min^−1^.

(6F*f*_*r*_), 8 breaths min^−1^. (8F*f*_*r*_), optimisation algorithm dynamic breathing frequency (Df_r_); heart rate (f_c_; beats min^−1^), respiratory sinus arrhythmia (RSA; s); within inspiration difference (Δi), within expiration difference (Δe), inter-breath phase peak-valley difference (ΔPV); Significantly different from Sf_r_ (*), 8F*f*_*r*_ (¥), Df_r_ (¤), 6F*f*_*r*_ (†), 4F*f*_*r*_ (§); P < 0.05.

The amplitude of RSA was significantly lower for the pregnant women, compared with non-pregnant women for all breathing conditions (p < 0.001). RSA during SDB for pregnant women increased to a level similar to that observed during spontaneous breathing (S*f*_*r*_) for non-pregnant women (Figure 3) and plateaued (saturated) at 6 breaths^.^ min^−1^ (6F*f*_*r*_) for both groups. The maximum amplitude of RSA during SDB (≤6 breaths^.^ min^−1^) was 2.1 times higher than RSA during S*f*_*r*_ for both the non-pregnant and pregnant group, albeit lower in absolute terms for pregnant women.

**FIGURE 3:**
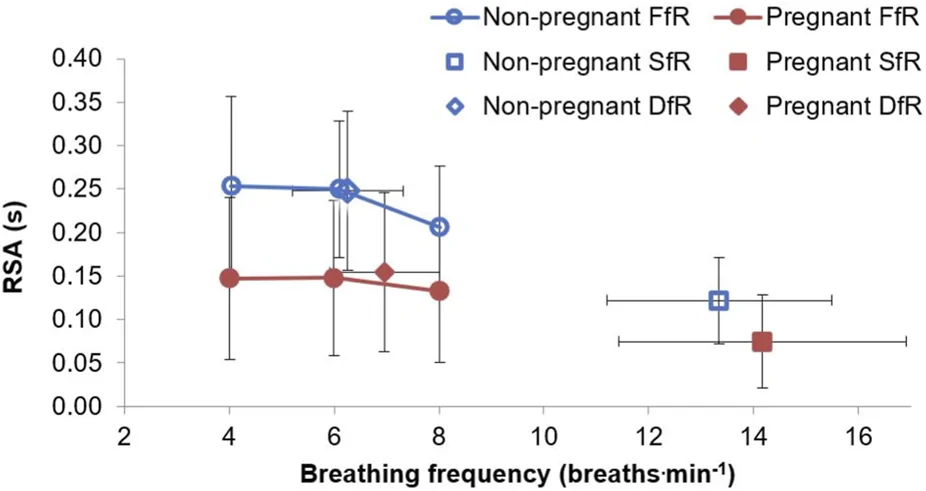
Respiratory sinus arrhythmia (RSA) response to slow and deep breathing. Data represent mean ± SD (non-pregnant n = 18, pregnant n = 17); Respiratory sinus arrhythmia (RSA); Fixed breathing frequency (Ff_r_), Spontaneous breathing (Sf_r_), optimisation algorithm dynamic breathing frequency (Df_r_).

### Preferred breathing condition

Fifty five percent of pregnant participants preferred the 6F*f*_*r*_ condition. Additionally, another 4 participants chose 6F*f*_*r*_ as their second preferred condition, where they had little preference between 2 conditions as their favourite. Therefore, a total of 78% of participants chose 6F*f*_*r*_ as their first or close second choice. There was no correlation between preferred breathing frequency and gestational age (r_s_(16) = −0.13, p = 0.623).

### Arterial blood pressures

The within-breath phase (maximum minus minimum values) peak-valley amplitude of SBP and DBP were significantly greater during both inspiration (Δi) and expiration (Δe) for all SDB conditions compared with spontaneous breathing (S*f*_*r*_; Table 4; p < 0.001). This was true for both pregnant and non-pregnant women. The only significant difference in SBPΔPV between pregnant and non-pregnant groups was for the 6F*f*_*r*_ condition (p = 0.001).

**TABLE 4:** Peak-valley differences (±SD) for blood pressure variables (mmHg).

Peak-valley differences	Group	Sf_r_	8F*f*_*r*_	Df_r_	6F*f*_*r*_	4F*f*_*r*_	Effect of condition *P* value	Group difference P value
SBP Δi	NP	3.6 ± 1.7^¥¤†§^	8.2 ± 2.9*^¤†§^	12.8 ± 5.4*^¥^	13.5 ± 4.6*^¥^	15.5 ± 6.1*^¥^	<0.001	0.925
	P	3.4 ± 1.5^¥¤†§^	9.1 ± 2.6*^¤†^	11.2 ± 3.7*^¥^	11.7 ± 3.8*^¥^	12.9 ± 4.9*	<0.001	
SBP Δe	NP	4.5 ± 2.5^¥¤†§^	6.9 ± 2.7*^¤†§^	10.2 ± 4.6*^¥^	10.5 ± 4.6*^¥^	12.1 ± 6.6*^¥^	<0.001	0.592
	P	3.7 ± 1.6^¥¤†§^	7.4 ± 2.5*^¤†^	9.0 ± 2.4*^¥^	9.7 ± 3.0*^¥^	11.7 ± 6.1*	<0.001	
SBP ΔPV	NP	−8.6 ± 3.6	−13.1 ± 7.0^§^	−11.5 ± 13.6^§^	−15.3 ± 9.5^§^	2.2 ± 18.4^¥¤†^	0.001	0.005
	P	−7.7 ± 2.6^§^	−8.5 ± 9.0^§^	−3.7 ± 13.1^§^	0.8 ± 14.7^§^	12.3 ± 9.8*^¥¤†^	<0.001	
SBP ΔPV_Ind	NP	15.0 ± 6.1	17.5 ± 5.6	19.1 ± 6.3	19.0 ± 5.1	17.4 ± 6.9	0.014	0.089
	P	12.9 ± 4.7^§^	13.9 ± 3.7	15.9 ± 3.1	15.9 ± 3.8	17.6 ± 6.0*	0.033	
DBP Δi	NP	2.4 ± 1.2^¥¤†§^	5.2 ± 1.8*^¤†§^	8.8 ± 2.9*^¥^	9.3 ± 3.1*^¥^	10.0 ± 3.0*^¥^	<0.001	0.118
	P	1.8 ± 0.8^¥¤†§^	6.6 ± 2.2*	7.7 ± 2.7*	7.5 ± 3.5*	7.6 ± 3.1*	<0.001	
DBP Δe	NP	3.2 ± 1.6^¥¤†§^	5.6 ± 2.1*^¤†§^	7.6 ± 3.1*^¥^	8.2 ± 3.2*^¥^	8.8 ± 3.1*^¥^	<0.001	0.553
	P	2.7 ± 1.3^¥¤†§^	6.7 ± 2.2*^†^	7.6 ± 1.8*	8.3 ± 2.2*^¥^	9.9 ± 4.0*	<0.001	
DBP ΔPV	NP	−4.0 ± 2.2^¥§^	−7.6 ± 2.0*^§^	−1.9 ± 11.1^§^	−3.4 ± 11.1^§^	11.6 ± 7.3*^¥¤†^	<0.001	0.097
	P	−4.6 ± 1.7^†§^	−6.1 ± 6.7^†§^	0.7 ± 9.3^§^	5.3 ± 9.1*^¥^	11.4 ± 3.4*^¥¤^	<0.001	
DBP ΔPV_Ind	NP	9.9 ± 4.4	10.6 ± 2.9	12.1 ± 3.3	12.1 ± 2.5	12.7 ± 3.3	0.014	0.130
	P	7.9 ± 2.8^†§^	9.7 ± 2.8	10.3 ± 2.4	10.4 ± n2.7*	12.8 ± 5.5*	0.005	

Data represent mean ± SD. Non-pregnant (NP) n = 18, Pregnant (P) n = 17. Spontaneous breathing (Sf_r_), fixed breathing frequency of 4 breaths min^−1^ (4F*f*_*r*_), 6 breaths min^−1^.

(6F*f*_*r*_), 8 breaths min^−1^. (8F*f*_*r*_), optimisation algorithm dynamic breathing frequency (Df_r_); systolic blood pressure (SBP; mmHg), diastolic blood pressure (DBP; mmHg); within inspiration difference (Δi), within expiration difference (Δe), inter-breath phase peak-valley difference (ΔPV), breath phase independent peak-valley difference (ΔPV_Ind); Significantly different from Sf_r_ (*), 8F*f*_*r*_ (¥), Df_r_ (¤), 6F*f*_*r*_ (†), 4F*f*_*r*_ (§); P < 0.05.

A high correlation (>0.8) was observed between SBPΔi and mean SBP and between SBPΔe and mean SBP, as well as for DBP equivalents, across all breathing conditions. To reveal the change in the amplitude of BP oscillations relative to mean BP, percentage change BP oscillations were calculated during each breath phase (peak-valley difference, Δi or Δe) as a percentage of average BP during corresponding inspiration or expiration (Figure 4). Values (%SBPΔi, %DBPΔi, %SBPΔe, %DBPΔe) for all SDB conditions were significantly different to S*f*_*r*_ for both pregnant and non-pregnant women. There were no significant differences between population groups.

**FIGURE 4:**
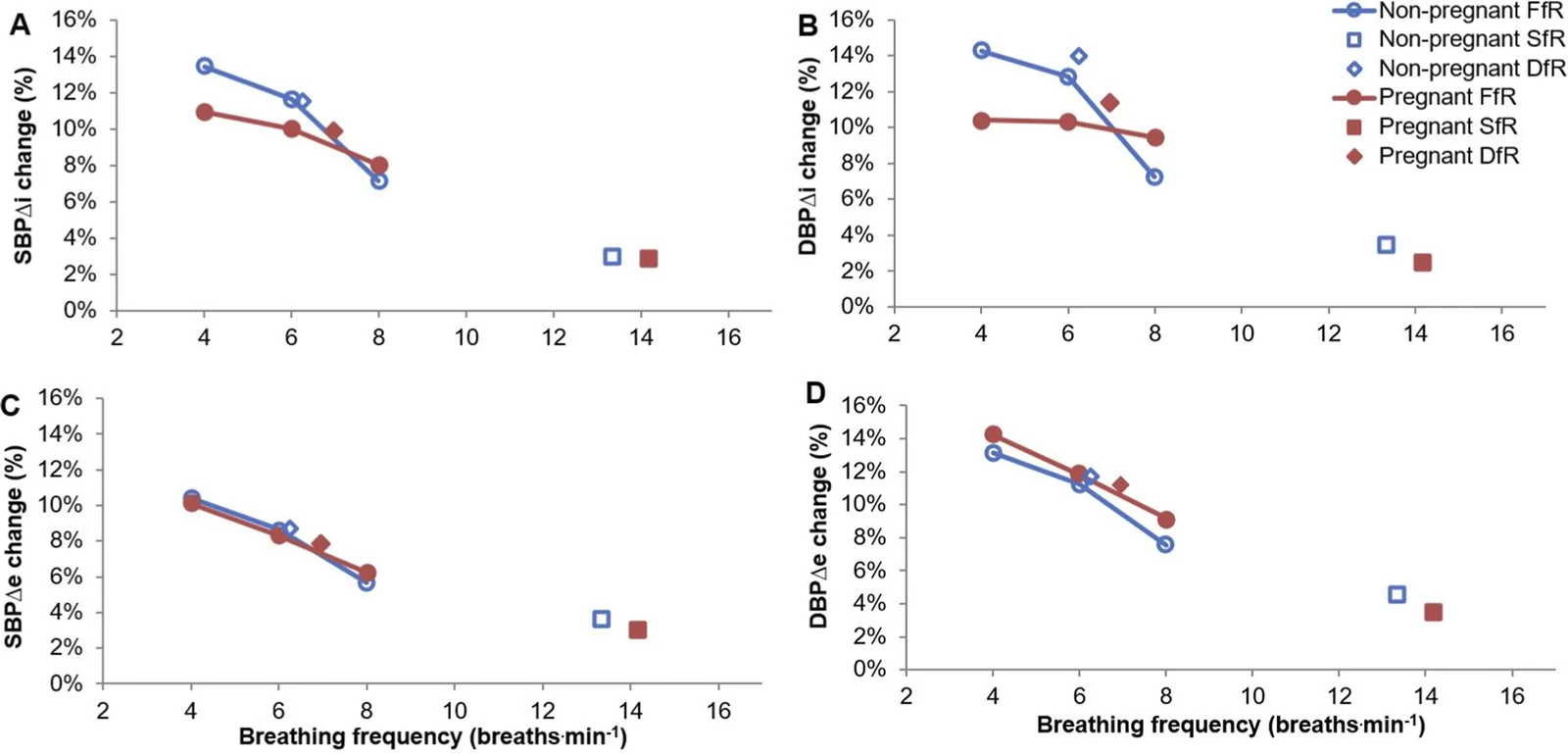
Blood pressure oscillations: Relative change for systolic blood pressure of Δi **(A)**, Δe **(B)** and diastolic blood pressure of Δi **(C)**, Δi **(D)**. Data represent mean (non-pregnant n = 18, pregnant n = 17); Systolic blood pressure (SBP), diastolic blood pressure (DBP); within expiration difference (Δi); within expiration difference (Δe); Fixed breathing frequency (Ff_r_), Spontaneous breathing (Sf_r_), optimisation algorithm dynamic breathing frequency (Df_r_). Variable calculated as SBPΔi as a percentage of average SBP during inspiration, or equivalent during expiration and for DBP.

Antenatal appointment recorded BP data (available for 58.8% of participants) revealed that no pregnant participants who submitted data developed hypertension [defined as SBP >140 mmHg and/or DBP >90 mmHg (NICE: National Institute for Health and Care Excellence, 2019)] following study participation.

### Stroke volume, cardiac output and total peripheral resistance

Peak-valley amplitude for SV and Q̇ during inspiration and expiration (SVΔi, SVΔe, Q̇Δi, Q̇Δe) were significantly higher during all SDB conditions compared with S*f*_*r*_ for non-pregnant and pregnant participants (Table 5). The only exception was SVΔi, which was not significantly different between 8F*f*_*r*_ and S*f*_*r*_ for non-pregnant women. Peak-valley SV was significantly lower for pregnant women compared with non-pregnant women during all SDB conditions, but not during S*f*_*r*_ (p < 0.005). Table 6 shows a significant increase in TPRΔi and TPRΔe during SDB compared with S*f*_*r*_ for both groups.

**TABLE 5:** Peak-valley differences (±SD) for stroke volume (SV) and cardiac output (Q̇).

Peak-valley differences	Group	Sf_r_	8F*f*_*r*_	Df_r_	6F*f*_*r*_	4F*f*_*r*_	Effect of condition *P* value	Group difference P value
SVΔi	NP	5.1 ± 2.0^¤†§^	7.3 ± 3.9^§^	7.1 ± 3.5*^§^	7.7 ± 4.0*^§^	11.7 ± 4.9*^¥¤†^	<0.001	0.014
	P	5.8 ± 2.0^¥¤†§^	9.0 ± 2.8*^†§^	10.7 ± 3.7*^§^	11.0 ± 3.3*^¥§^	15.3 ± 5.0 *^¥¤†^	<0.001	
SVΔe	NP	5.6 ± 2.0^¥¤†§^	8.2 ± 3.1*^§^	8.9 ± 4.0*^§^	9.2 ± 3.9*	13.5 ± 6.3*^¥¤^	<0.001	0.827
	P	5.2 ± 1.9^¥¤†§^	7.9 ± 2.3*^§^	9.2 ± 2.3*^§^	9.6 ± 2.3*^§^	12.5 ± 3.7 *^¥¤†^	<0.001	
SV ΔPV	NP	−8.9 ± 5.5^§^	−13.1 ± 8.0^§^	−13.8 ± 8.5	−14.6 ± 7.6	−18.6 ± 6.9*^¥^	0.001	0.002
	P	−8.0 ± 6.5	−2.9 ± 12.1	−3.4 ± 15.1	−1.7 ± 14.8	−0.4 ± 17.7	0.119	
SVΔPV _Ind	NP	11.9 ± 4.2^§^	13.7 ± 5.9	13.6 ± 6.4	13.4 ± 6.4	16.2 ± 6.4*	0.027	0.361
	P	13.0 ± 4.0^§^	13.4 ± 4.1^§^	15.5 ± 5.6	15.6 ± 4.6	18.0 ± 5.2*^¥^	0.002	
Q̇Δi	NP	292 ± 119^¥¤†§^	1092 ± 646*	1107 ± 561*	1129 ± 607*	1073 ± 474*	<0.001	0.063
	P	586 ± 293^¥¤†§^	1151 ± 348*	1340 ± 468*	1292 ± 422*	1453 ± 300*	<0.001	
Q̇Δe	NP	414 ± 184^¥¤†§^	831 ± 358*	840 ± 469*	787 ± 424*	747 ± 413*	<0.001	0.018
	P	563 ± 265^¥¤†§^	920 ± 243*	1012 ± 336*	1040 ± 355*	1164 ± 479*	<0.001	
Q̇ ΔPV	NP	−738 ± 203^¥¤†§^	−638 ± 1153^¤†§^	402 ± 1268*¥	492 ± 1208*^¥^	903 ± 923*^¥^	<0.001	<0.001
	P	−745 ± 663^¥¤†§^	1181 ± 732*	1432 ± 870*	1431 ± 842*	1452 ± 901*	<0.001	
Q̇ΔPV _Ind	NP	1352 ± 1982	1302 ± 667	1366 ± 1038	1191 ± 565	1018 ± 540	0.489	0.409
	P	1198 ± 341	1382 ± 302	1602 ± 553	1496 ± 373	1426 ± 282	0.006	

Data represent mean ± SD. Non-pregnant (NP) n = 18, Pregnant (P) n = 17. Spontaneous breathing (Sf_r_), fixed breathing frequency of 4 breaths min^−1^ (4F*f*_*r*_), 6 breaths min^−1^.

(6F*f*_*r*_), 8 breaths min^−1^. (8F*f*_*r*_), optimisation algorithm dynamic breathing frequency (Df_r_); stroke volume (SV; ml), cardiac output (Q̇; ml. min^−1^); within inspiration difference (Δi), within expiration difference (Δe), inter-breath phase peak-valley difference (ΔPV); breath phase independent peak-valley difference (ΔPV_Ind). Significantly different from Sf_r_ (*), 8F*f*_*r*_ (¥), Df_r_ (¤), 6F*f*_*r*_ (†), 4F*f*_*r*_ (§); P < 0.05.

**TABLE 6:** Peak-valley differences (±SD) for total peripheral resistance (TPR; *mmHg* ^*.*^ *min* ^*.*^ *L* ^*−1*^) and pulse wave velocity (PWV; *m* ^*.*^ *s* ^*-1*^) variables.

Peak-valley differences	Group	Sf_r_	8F*f*_*r*_	Df_r_	6F*f*_*r*_	4F*f*_*r*_	Effect of condition *P* value	Group difference P value
TPR Δi	NP	1.8 ± 2.2^¥†§^	4.0 ± 1.9*	3.0 ± 1.1	4.3 ± 2.4*	3.5 ± 1.7*	<0.001	0.001
	P	1.1 ± 0.6^¥¤†§^	1.8 ± 0.8*	2.0 ± 0.8*	2.0 ± 0.9*	2.2 ± 0.7*	<0.001	
TPR Δe	NP	2.1 ± 2.0^†^	3.1 ± 1.4	2.8 ± 1.4	3.7 ± 2.3*	3.2 ± 1.3	0.020	0.006
	P	1.1 ± 0.6^¤†§^	1.7 ± 1.1^†^	2.0 ± 1.2*	2.2 ± 1.1*^¥^	2.2 ± 1.0*	<0.001	
TPR ΔPV	NP	2.3 ± 3.8^¤†^	0.8 ± 4.7	−3.2 ± 2.7*	−3.3 ± 5.1*	0.3 ± 4.8	<0.001	0.115
	P	0.4 ± 1.7^¥¤†^	−2.8 ± 1.3*	−2.9 ± 1.2*	−2.5 ± 1.7*	−0.7 ± 2.7	<0.001	
TPRΔPV _Ind	NP	6.0 ± 6.3	5.2 ± 2.1	4.4 ± 1.9	5.4 ± 2.7	4.1 ± 1.7	0.192	0.002
	P	2.5 ± 1.1	3.0 ± 1.4	2.9 ± 1.2	2.8 ± 1.2	2.6 ± 1.1	0.159	
PWVΔi	NP	0.2 ± 0.1^¥¤†§^	0.4 ± 0.2*	0.5 ± 0.2*	0.5 ± 0.2*	0.4 ± 0.1*	<0.001	0.194
	P	0.2 ± 0.1^¥¤†§^	0.6 ± 1.3*^†§^	0.3 ± 0.1*	0.4 ± 0.2*^¥^	0.5 ± 0.2*^¥^	<0.001	
PWVΔe	NP	0.3 ± 0.1^¥¤†^	0.4 ± 0.2*	0.4 ± 0.2*	0.4 ± 0.2*	0.4 ± 0.1	0.001	0.528
	P	0.3 ± 0.1	0.3 ± 0.1	0.4 ± 0.4	0.3 ± 0.1	0.5 ± 0.2	0.088	
PWV ΔPV	NP	0.4 ± 0.1^¥¤†§^	−0.7 ± 0.5*^†^	−0.6 ± 0.3*^†^	0.6 ± 0.3*^¥†§^	−0.3 ± 0.5*^†^	<0.001	0.002
	P	−0.3 ± 0.2	0.0 ± 1.4	−0.4 ± 0.5	−0.1 ± 0.5	−0.1 ± 0.6	0.158	

Data represent mean ± SD. Non-pregnant (NP) n = 18, Pregnant (P) n = 17. Spontaneous breathing (Sf_r_), fixed breathing frequency of 4 breaths min^−1^ (4F*f*_*r*_), 6 breaths min^−1^.

(6F*f*_*r*_), 8 breaths min^−1^. (8F*f*_*r*_), optimisation algorithm dynamic breathing frequency (Df_r_); total peripheral resistance (TPR; mmHg.min.L^-1^), pulse wave velocity (PWV; m.s^-1^); within inspiration difference (Δi), within expiration difference (Δe), inter-breath phase peak-valley difference (ΔPV), breath phase independent peak-valley difference (ΔPV_Ind). Significantly different from Sf_r_ (*), 8F*f*_*r*_ (¥), Df_r_ (¤), 6F*f*_*r*_ (†), 4F*f*_*r*_ (§); P < 0.05.

## Discussion

The present study builds on work from Felton et al. (2026) to characterise acute cardiovascular responses to SDB, including an analysis of the inter- and intra-breath phase perturbations created by breathing, taking a Network Physiology approach. This work compares these acute cardiovascular responses in pregnant and non-pregnant women for the first time. The study showed that SDB can increase RSA in pregnant women to levels similar to the RSA observed during spontaneous breathing for non-pregnant participants, revealing the ability of SDB to return RSA to pre-pregnancy levels.

Although heart rate was higher in the pregnant group, their RSA was significantly lower for all breathing conditions, measured at just 58% of the value observed in non-pregnant women during spontaneous breathing (S*f*_*r*_). This observation is consistent with the 65% difference found by Miyazato and Matsukawa (2010). SDB caused a significant increase in RSA for both non-pregnant and pregnant women compared with spontaneous breathing (S*f*_*r*_); relative RSA (maximum RSA compared with baseline RSA) increased by a maximum of 48% and 47%, respectively. Therefore, although absolute maximum RSA was higher in the non-pregnant group (0.25 v. 0.15 s), the response to SDB created an almost 50% increase in the amplitude of RSA in both groups. This is consistent with the absolute RSA response to relaxation, which was also lower in pregnant women compared with non-pregnant women (DiPietro et al., 2012). Thus, during SDB breathing, the present study found a similar relative (%) increase in RSA amplitude, despite a lower absolute RSA amplitude. This shows that despite the altered baseline state of the physiological network caused by pregnancy (evidenced by the lower absolute RSA), the responsiveness of the network to SDB and the coupling of the respiratory and cardiovascular systems is maintained during pregnancy.

As an attenuated RSA has been suggested as a biophysical marker of pre-eclampsia (Lakhno, 2016), the ability of an intervention to increase RSA during pregnancy is promising. Attenuated RSA *per se* is not the cause of hypertension, but reflects the functional state of the autonomic nervous system (Buchner, 2018), and as there is an overactivity of the sympathetic arm during gestational hypertension, changes in RSA may reflect an improvement in the balance of the autonomic nervous system. Although it should be noted that RSA’s ability to reflect the autonomic nervous system is queried (see point: counterpoint series) (Julien et al., 2009; Karemaker, 2009a; Karemaker, 2009b; Eckberg, 2009), and therefore any suggestions of relationships must be carefully interpreted. Whether SDB can increase long-term RSA in pregnant women after daily SDB practice is unknown but needs investigating.

### Effect of response kinetics and comparison of pregnant and non-pregnant women’s responses

The amplitude of BP oscillations (both SBP and DBP) during inspiration and expiration increased as breathing frequency reduced, reaching a peak at 4 breaths^.^ min^−1^ (4F*f*_*r*_), which was up to 4 and a half times higher (14.3%, 10 mmHg) than during spontaneous breathing (S*f*_*r*_ *;* see Figure 4). This supports previous research (Felton et al., 2026) which suggested that the amplitude of BP oscillations may be further increased by breathing at frequencies below 6 breaths^.^ min^−1^. However, although the amplitude of BP oscillations was further increased below 6 breaths^.^ min^−1^, there was no significant difference at 4F*f*_*r*_ from the response during 6F*f*_*r*_ suggesting minimal differences between SDB conditions. The difference was only an average 1.2 mmHg (±0.7 mmHg) between 4F*f*_*r*_ and 6F*f*_*r*_, which is unlikely to produce a meaningful clinical difference between the two conditions if used as part of a long-term SDB intervention. These lower breathing frequencies may represent a great synchronisation between respiratory cycles and cardiovascular responses (as also evidenced by RSA), indicating that the larger fluctuations in cardiovascular variables found at lower breathing frequencies may be a result of stronger multi-system coordination, with near maximal network synchronisation occurring around 6 breaths^.^ min^−1^.

The initial set of analyses show that heart rate, stroke volume and cardiac output were significantly higher in pregnant women during spontaneous breathing (S*f*_*r*_), which is in agreement with the known adaptations caused by pregnancy (Sanghavi and Rutherford, 2014). Pregnant women had higher cardiac output and stroke volume at equivalent breathing frequencies, compared with non-pregnant women, which is also consistent with the higher cardiovascular response seen during aerobic exercise in pregnant women (Hegewald and Crapo, 2011). Total peripheral resistance was significantly lower in pregnant women compared with non-pregnant women in the present study, which is most likely attributable to vasodilation that occurs during pregnancy (Ngene and Moodley, 2017). Levels of BP are reliant on the balance between total peripheral resistance and cardiac output. Therefore, to maintain BP during pregnancy, cardiac output is increased to counteract the decreased total peripheral resistance (Moser et al., 2012). In the present study there was no significant differences in BP between pregnant and non-pregnant women, despite the lower total peripheral resistance, due to a significantly higher cardiac output in the pregnant women group.

Interestingly, although an increased tidal volume was expected in pregnant women (McAuliffe et al., 2002), this was only observed during spontaneous breathing (S*f*_*r*_), where tidal volume was double that of non-pregnant women. There were no significant differences in tidal volume between non-pregnant and pregnant women during any SDB conditions, suggesting an ability of the respiratory system of pregnant women to adapt comfortably to reduced breathing frequencies in a similar way to non-pregnant women. Tidal volume in the pregnant women group increased significantly as breathing frequency was reduced below 8 breaths^.^ min^−1^. This also supports the suggestion that despite differences in baseline cardiovascular variables in pregnant women, the responsiveness of the physiological network is maintained during pregnancy, and mimics responses seen in non-pregnant women.

### Implications of findings for optimal breathing frequency and methods of implementation for clinical SDB interventions

Although there were limited differences in the cardiovascular responses observed in the present study between the SDB conditions of 4F*f*_*r*_ 6F*f*_*r*_ and D*f*_*r*_, for many variables (such as percentage amplitudes of BP oscillations) the 8F*f*_*r*_ condition did not deliver a significantly different cardiovascular response compared with spontaneous breathing (S*f*_*r*_). This suggests that 8 breaths^.^ min^−1^ may be too high a breathing frequency to elicit the full cardiovascular response of SDB. Previous research found differences in heart rate variability when breathing frequencies of 6.5 and 7 breaths^.^ min^−1^ were used compared with lower breathing frequencies, but only in one variable (RMSSD), with no significant differences between SDB frequencies in low frequency heart rate variability (You et al., 2024). This may have consequences for the breathing frequency chosen as part of long-term interventions and further supports the theory that 6 breaths^.^ min^−1^ may be the optimal SDB frequency to maximise haemodynamic changes (Russo et al., 2017). RESPeRATE is a device that has been used to deliver SDB daily with the aim of reducing chronic BP and aims to reduce breathing frequency to <10 breaths^.^ min^−1^. Previous research measured an average breathing frequency of 8.1 breaths^.^ min^−1^ for the first 5-min of RESPeRATE use (Felton et al., 2026), which could limit the effectiveness of the SDB if the maximum perturbations are not achieved for the full 10 min SDB session. However, breathing frequencies within mindfulness-based breathing exercises delivered at approx. 10 breaths^.^ min^−1^ have still produced a significant reduction in blood pressure compared with control groups during pregnancy (Kılıçlı and Simge, 2025).

The preferred breathing frequency of the eighteen pregnant women was 6 breaths^.^ min^−1^ (6F*f*_*r*_), when asked which breathing frequency they would prefer to undertake if they were to continue with the SDB exercise daily until birth. There was no correlation between gestational age and preferred SDB frequency, or for optimal breathing frequency derived from the optimisation algorithm, suggesting that all SDB frequencies are manageable at all stages of gestation. Therefore, the present study suggests that future studies should utilise 6 breaths^.^ min^−1^ (6F*f*_*r*_) for SDB interventions with pregnant women, which provides a good agreement between optimising physiological responses and participant preference.

### Strengths, limitations and future research

When examining the response and adaptations of the physiological systems to internal and external perturbations it is important to understand the system interactions (Ivanov, 2021), and when examining the response to the external perturbation of SDB, the dynamic interactions between the respiratory, cardiac and vascular systems are only revealed when analysing intra- and inter-breath cardiovascular oscillations. Therefore, this study reiterates the importance of examining beyond mean cardiovascular values when examining responses to SDB, as when only mean values are examined (Supplementary Material) the results mask the complex coordination of the physiological systems including the increase in the amplitude of oscillations that occur to maintain homeostasis during SDB.

However, it must be acknowledged that haemodynamic changes in this study were measured non-invasively, and as such all cardiovascular variables are estimates of the true responses. Nonetheless, the estimation of SV using the Modelflow method (Finapres) has previously been shown to have excellent agreement with SV measured by Doppler ultrasound (Van Lieshout et al., 2003) and when blood is withdrawn by phlebotomy (Leonetti et al., 2004). The Finapres BP measures also correlate highly (r = 0.67, p < 0.001) with auscultatory BP measurements in normotensive participants (Carlson et al., 2019). However, when considering accuracy of measurements during SDB, the use of indirect measurement needs to be acknowledged.

Additionally, the sample size for each group was relatively small for examining differences between groups, particularly when investigating variables which have high variability, and for sub-group analysis such as potential changes in the cardiovascular response across gestational age. Future research should recruit more participants across different gestational ages. There is also some evidence that cardiac autonomic functioning may differ across the menstrual cycle (Delcourt et al., 2025), although no differences in responses to SDB for oral contraceptive use and menstrual phase were found (Nili et al., 2017). Menstrual cycle phase or oral contraceptive use was not recorded or controlled for in this study, but future research should record menstrual cycle phase and/or contraceptive use to ensure differences are explored. It will also be important for future research to examine if cardiovascular responses to SDB change in a hypertensive pregnant population. It has previously been found that within non-pregnant individuals responses differ between normotensive and hypertensive individuals (Joseph et al., 2005), so this study needs to be replicated in women diagnosed with gestational hypertension.

## Conclusion

The present study adds to growing evidence that the analysis of inter- and intra-breath phase haemodynamic oscillations are vital to reveal the full extent of the cardiovascular perturbations created by SDB. The cardiovascular responses to SDB are similar in healthy pregnant and healthy non-pregnant women, with no significant differences in relative amplitude of BP oscillations and a similar relative increase in RSA from baseline values (S*f*_*r*_). RSA is attenuated during spontaneous breathing in pregnant women, but can be increased acutely to non-pregnant levels by SDB. When designing interventions utilising SDB it is important to consider that a breathing frequency of ≤6 breaths^.^ min^−1^ is needed to induce maximal cardiovascular perturbations, and that aiming for SDB ≤10 breaths^.^ min^−1^ is not sufficient. The data support future studies investigating the long-term changes to RSA, BP and other cardiovascular variables following daily practice of SDB using a breathing frequency of 6 breaths^.^ min^−1^ with pregnant women.

## Data Availability

The original contributions presented in the study are included in the article/Supplementary Material, further inquiries can be directed to the corresponding author.
