# Supplementary material for: Acute cardiovascular responses to slow and deep breathing in normotensive non-pregnant and pregnant women
Source: Front Netw Physiol. 2026 Oct 14;6:1913383. doi: 10.3389/fnetp.2026.1913383 (PMC13713283; doi:10.3389/fnetp.2026.1913383)
Supplement: Supplementary file 1 [file Table1.docx]

Supplementary Material: Mean Cardiovascular Data

## Acute cardiovascular responses to slow and deep breathing in normotensive non-pregnant and pregnant women

|  |  | **S*f_r_*** | **8F*f_r_*** | **D*f_r_*** | **6F*f_r_*** | **4F*f_r_*** | **Effect of condition**  ***P* value** | | **Group difference**  **P value** |
| --- | --- | --- | --- | --- | --- | --- | --- | --- | --- |
| *F_c_* | *NP* | 67.6 ± 10.9 | 70.7 ± 11.6 | 70.0 ± 11.0 | 70.0 ± 10.5 | 69.5 ± 10.3 | 0.091 | | *0.001* |
|  | *P* | 80.7 ± 10.1 | 82.6 ± 9.8 | 80.3 ± 9.3 | 81.7 ± 8.6 | 79.9 ± 8.4 | 0.123 | |  |
| *f_c_ i* | *NP* | 67.0 ± 11.3*^¤†§^* | 72.5 ± 12.1*^†^* | 74.4 ± 12.5* | 74.4 ± 12.0**^¥^* | 74.5 ± 11.0* | <0.001 | | *0.003* |
|  | *P* | 80.4 ± 10.5*^¥¤†^* | 85.2 ± 10.0* | 83.9 ± 8.8* | 85.1 ± 7.5* | 82.1 ± 7.3 | <0.001 | |  |
| *f_c_ e* | *NP* | 68.3 ± 10.6*^§^* | 68.5 ± 10.8*^†§^* | 65.8 ± 10.3 | 65.6 ± 9.2*^¥^* | 64.6 ± 10.1**^¥^* | 0.002 | | *0.001* |
|  | *P* | 80.9 ± 9.8 | 79.7 ± 9.8 | 76.4 ± 10.1 | 78.1 ± 10.0 | 77.7 ± 9.9 | 0.009 | |  |
| *f_c_ Δ* | *NP* | -1.4 ± 1.4*^¥¤†§^* | 4.0 ± 2.9**^¤†§^* | 8.6 ± 5.4**^¥^* | 8.9 ± 4.9**^¥^* | 9.9 ± 4.8**^¥^* | <0.001 | | *0.240* |
|  | *P* | -0.5 ± 2.4*^¥¤†^* | 5.5 ± 3.3* | 7.4 ± 4.7* | 7.0 ± 5.0* | 4.3 ± 5.1 | <0.001 | |  |
| *SV* | *NP* | 67.4 ± 19.8 | 66.7 ± 21.1 | 68.3 ± 21.3 | 66.2 ± 22.0 | 68.5 ± 18.4 | | 0.539 | *0.008* |
|  | *P* | 82.3 ± 9.8 | 81.4 ± 10.4 | 82.5 ± 10.0 | 82.7 ± 9.2 | 83.1 ± 10.2 | | 0.920 |  |
| *SVi* | *NP* | 65.2 ± 19.7 | 63.5 ± 20.4 | 64.9 ± 20.4 | 63.1 ± 20.7 | 65.1 ± 18.1 | | 0.366 | *0.002* |
|  | *P* | 80.3 ± 9.5 | 80.9 ± 10.5 | 81.9 ± 9.9 | 82.3 ± 9.2 | 82.7 ± 10.0 | | 0.640 |  |
| *SVe* | *NP* | 69.6 ± 20.0 | 69.9 ± 21.9 | 71.6 ± 22.4 | 69.3 ± 23.3 | 71.9 ± 18.9 | | 0.490 | *0.026* |
|  | *P* | 84.2 ± 10.3 | 81.8 ± 10.8 | 83.0 ± 10.8 | 83.1 ± 9.9 | 83.5 ± 10.7 | | 0.854 |  |
| *SVΔ* | *NP* | -4.4 ± 2.9 | -6.4 ± 3.6 | -6.7 ± 4.6 | -6.2 ± 4.4 | -6.8 ± 3.5 | | 0.188 | *<0.001* |
|  | *P* | -3.9 ± 3.0 | -0.9 ± 4.2 | -1.1 ± 5.7 | -0.8 ± 4.9 | -0.7 ± 4.2 | | 0.027 |  |
| *Q̇* | *NP* | 4441 ± 1047 | 4564 ± 1144 | 4633 ± 1097 | 4482 ± 1220 | 4632 ± 1017 | | 0.530 | *<0.001* |
|  | *P* | 6596 ± 923 | 6652 ± 1075 | 6564 ± 981 | 6706 ± 1027 | 6573 ± 863 | | 0.728 |  |
| *Q̇i* | *NP* | 4247 ± 1018*^¤^* | 4476 ± 1181 | 4707 ± 1184* | 4562 ± 1266 | 4737 ± 1096 | | 0.026 | *<0.001* |
|  | *P* | 6427 ± 953*^¥¤†^* | 6858 ± 1090* | 6855 ± 1034* | 6995 ± 990* | 6749 ± 884 | | 0.001 |  |
| *Q̇e* | *NP* | 4639 ± 1083 | 4639 ± 1117 | 4566 ± 1034 | 4406 ± 1197 | 4527 ± 952 | | 0.435 | *<0.001* |
|  | *P* | 6765 ± 913*^¤§^* | 6447 ± 1077 | 6268 ± 958* | 6427 ± 1079 | 6399 ± 864* | | 0.003 |  |
| *Q̇Δ* | *NP* | -392 ± 155*^¥¤†§^* | –163 ± 250**^¤†§^* | 141 ± 307**^¥^* | 156 ± 278**^¥^* | 210 ± 264**^¥^* | | <0.001 | *<0.001* |
|  | *P* | -339 ± 286*^¥¤†§^* | 411 ± 278**^¤^* | 588 ± 351**^¥^* | 568 ± 238**^§^* | 350 ± 257**^†^* | | <0.001 |  |

Table S1: Mean values (±SD) and inter-breath differences (Δ) for heart rate (*f_c_*), stroke volume (SV) and cardiac output (Q̇) variables

*Data represent mean ± SD. Non-pregnant (NP) n=18, Pregnant (P) n=17. Spontaneous breathing (Sf_r_), fixed breathing frequency of 4 breaths^.^minute^-1^ (4Ff_r_), 6 breaths^.^minute^-1^ (6Ff_r_), 8 breaths^.^minute^-1^ (8Ff_r_), optimisation algorithm dynamic breathing frequency (Df); heart rate (f_c_; beats^.^min^-1^), stroke volume (SV; ml), cardiac output (*Q̇; *ml^.^min^-1^); mean inspiration (i), mean expiration (e), inter-breath difference (∆; i minus e); Significantly different from* S*f_r_* *(*), 8Ff_r_ (¥), Df_r_ (¤), 6Ff_r_ (†), 4Ff_r_ (§); P<0.05.*

|  |  | **S*f_r_*** | **8F*f_r_*** | **D*f_r_*** | **6F*f_r_*** | **4F*f_r_*** | **Effect of condition**  ***P* value** | **Group difference**  **P value** |
| --- | --- | --- | --- | --- | --- | --- | --- | --- |
| *SBP* | *NP* | 121.3 ± 17.6 | 118.0 ± 9.6 | 115.3 ± 19.9 | 119.8 ± 13.8 | 116.2 ± 10.5 | 0.481 | *0.921* |
|  | *P* | 122.1 ± 12.5 | 117.1 ± 17.4 | 115.4 ± 13.9 | 117.1 ± 10.7 | 117.2 ± 15.8 | 0.347 |  |
| *SBPi* | *NP* | 118.9 ± 17.4 | 114.3 ± 9.8 | 112.3 ± 20.2 | 117.1 ± 14.7 | 116.5 ± 10.4 | 0.475 | *0.681* |
|  | *P* | 119.8 ± 12.5 | 115.6 ± 18.1 | 114.9 ± 14.4 | 118.0 ± 11.5 | 118.7 ± 15.5 | 0.519 |  |
| *SBPe* | *NP* | 123.8 ± 17.8 | 121.8 ± 9.6 | 118.4 ± 20.0 | 122.7 ± 13.3 | 115.8 ± 11.1 | 0.200 | *0.538* |
|  | *P* | 124.3 ± 12.5 | 118.5 ± 16.8 | 116.0 ± 13.8 | 116.3 ± 10.3 | 115.7 ± 16.3 | 0.062 |  |
| *SBPΔ* | *NP* | –4.9 ± 2.1*^§^* | –7.5 ± 3.4*^§^* | –6.0 ± 5.0*^§^* | –5.6 ± 5.2*^§^* | 0.7 ± 5.0**^¥¤†^* | <0.001 | *0.001* |
|  | *P* | -4.5 ± 1.8*^¤†§^* | -2.9 ± 3.8*^†§^* | -1.1 ± 4.1**^†§^* | 1.7 ± 4.7**^¥¤^* | 3.0 ± 2.2**^¥¤^* | 0.000 |  |
| *DBP* | *NP* | 74.7 ± 17.1 | 73.1 ± 7.3 | 67.5 ± 14.7 | 74.1 ± 11.4 | 69.8 ± 8.7 | 0.166 | *0.942* |
|  | *P* | 74.5 ± 10.2 | 73.2 ± 12.8 | 69.7 ± 10.3 | 71.6 ± 7.3 | 71.3 ± 12.3 | 0.289 |  |
| *DBPi* | *NP* | 73.9 ± 17.3 | 71.8 ± 7.3 | 67.1 ± 15.4 | 73.6 ± 11.9 | 71.9 ± 9.0 | 0.286 | *0.846* |
|  | *P* | 73.2 ± 9.9 | 72.4 ± 13.2 | 69.9 ± 10.7 | 72.9 ± 7.9 | 72.9 ± 12.0 | 0.561 |  |
| *DBPe* | *NP* | 75.5 ± 17.0 | 74.3 ± 7.3 | 68.0 ± 14.2 | 74.6 ± 11.1 | 67.7 ± 8.6 | 0.064 | *0.961* |
|  | *P* | 75.8 ± 10.4*^¤†^* | 74.1 ± 12.4 | 69.5 ± 10.0* | 70.3 ± 7.0* | 69.7 ± 7.0 | 0.021 |  |
| *DBPΔ* | *NP* | –1.5 ± 1.0*^§^* | –2.5 ± 1.5*^§^* | –0.9 ± 3.4*^§^* | –1.0 ± 3.4*^§^* | 4.2 ± 3.3**^¥¤†^* | <0.001 | *0.134* |
|  | *P* | -2.5 ± 0.9*^¤†§^* | -1.7 ± 1.8*^¤†§^* | 0.3 ± 2.1**^¥†§^* | 2.6 ± 2.5**^¥¤^* | 3.2 ± 2.2**^¥¤^* | <0.001 |  |

Table S2 Mean values (±SD) and inter-breath differences (Δ) for blood pressure variables (mmHg)

*Data represent mean ± SD (mmHg). Non-pregnant (NP) n=18, Pregnant (P) n=17. Spontaneous breathing (Sf_r_), fixed breathing frequency of 4 breaths^.^minute^-1^ (4Ff_r_), 6 breaths^.^minute^-1^ (6Ff_r_), 8 breaths^.^minute^-1^ (8Ff_r_), optimisation algorithm dynamic breathing frequency (Df_r_); systolic blood pressure (SBP), diastolic blood pressure (DBP); mean inspiration (i), mean expiration (e), inter-breath difference (∆; i minus e); Significantly different from* S*f_r_* *(*), 8Ff_r_ (¥), Df_r_ (¤), 6Ff_r_ (†), 4Ff_r_ (§); P<0.05.*

Table S3 Mean values (±SD) and inter-breath differences (Δ) for total peripheral resistance (TPR) and pulse wave velocity (PWV) variables

|  |  | **S*f_r_*** | **8F*f_r_*** | **D*f_r_*** | **6F*f_r_*** | **4F*f_r_*** | **Effect of condition**  ***P* value** | **Group difference**  **P value** |
| --- | --- | --- | --- | --- | --- | --- | --- | --- |
| *TPR* | *NP* | 21.5 ± 8.3 | 20.0 ± 3.7 | 18.3 ± 3.5 | 20.9 ± 5.4 | 18.8 ± 3.5 | 0.171 | *<0.001* |
|  | *P* | 13.8 ± 3.1 | 13.4 ± 3.1 | 13.1 ± 2.6 | 13.1 ± 2.4 | 13.2 ± 2.2 | 0.419 |  |
| *TPRi* | *NP* | 22.2 ± 8.6 | 20.0 ± 3.9 | 17.8 ± 3.6 | 20.2 ± 5.1 | 18.9 ± 3.8 | 0.084 | *<0.001* |
|  | *P* | 14.0 ± 3.0*^¤†^* | 12.8 ± 2.9 | 12.5 ± 2.6* | 12.7 ± 2.3* | 13.1 ± 2.2 | 0.020 |  |
| *TPRe* | *NP* | 20.8 ± 7.9 | 20.0 ± 3.5 | 18.8 ± 3.6 | 21.6 ± 5.7 | 18.8 ± 3.4 | 0.233 | *<0.001* |
|  | *P* | 13.7 ± 3.2 | 14.0 ± 3.2 | 13.7 ± 2.7 | 13.5 ± 2.6 | 13.3 ± 2.3 | 0.600 |  |
| *TPRΔ* | *NP* | 1.3 ± 1.1*^¥¤†^* | 0.0 ± 1.1**^¤†^* | -1.0 ± 0.9**^¥^* | -1.4 ± 1.3**^¥^* | 0.0 ± 1.6 | <0.001 | *0.015* |
|  | *P* | 0.2 ± 0.7*^¥¤†^* | -1.2 ± 0.7**^§^* | -1.2 ± 0.6**^§^* | -0.8 ± 0.7**^§^* | -0.2 ± 0.4*^¥¤†^* | <0.001 |  |
| PWV | *NP* | 4.6 ± 0.4 | 4.6 ± 0.4 | 4.7 ± 0.4 | 4.7 ± 0.4 | 4.7 ± 0.4 | 0.143 | *0.014* |
|  | *P* | 5.1 ± 0.5 | 5.1 ± 0.5 | 5.0 ± 0.4 | 5.0 ± 0.5 | 5.0 ± 0.5 | 0.081 |  |
| PWVi | *NP* | 4.6 ± 0.4 | 4.5 ± 0.4*^§^* | 4.6 ± 0.4 | 4.6 ± 0.4 | 4.7 ± 0.4*^¥^* | 0.002 | *0.007* |
|  | *P* | 5.0 ± 0.5 | 5.0 ± 0.6 | 4.9 ± 0.4 | 5.0 ± 0.5 | 5.0 ± 0.5 | 0.205 |  |
| PWVe | *NP* | 4.7 ± 0.4 | 4.7 ± 0.4 | 4.8 ± 0.4 | 4.8 ± 0.4 | 4.8 ± 0.4 | 0.472 | *0.029* |
|  | *P* | 5.2 ± 0.5*^†§^* | 5.1 ± 0.5*^†^* | 5.1 ± 0.5 | 5.0 ± 0.5**^¥^* | 5.1 ± 0.5* | 0.004 |  |
| PWV*Δ* | *NP* | –0.2 ± 0.1 | –0.2 ± 0.1 | –0.1 ± 0.1 | –0.2 ± 0.1 | –0.1 ± 0.2 | 0.030 | *0.006* |
|  | *P* | -0.14 ± 0.1*^†^* | -0.10 ± 0.2*^†§^* | -0.11 ± 0.1*^†^* | -0.02 ± 0.1**^¥¤^* | -0.02 ± 0.1*^¥^* | <0.001 |  |

*Data represent mean ± SD. Non-pregnant (NP) n=18, Pregnant (P) n=17. Spontaneous breathing (Sf_r_), fixed breathing frequency of 4 breaths^.^minute^-1^ (4Ff_r_), 6 breaths^.^minute^-1^ (6Ff_r_), 8 breaths^.^minute^-1^ (8Ff_r_), optimisation algorithm dynamic breathing frequency (Df_r_); total peripheral resistance (TPR; mmHg⋅min⋅L^-1^, pulse wave velocity (PWV; m^.^s^-1)^; mean inspiration (i), mean expiration (e), inter-breath difference (∆; i minus e); Significantly different from* S*f_r_* *(*), 8Ff_r_ (¥), Df_r_ (¤), 6Ff_r_ (†), 4Ff_r_ (§); P<0.05.*
